# Advancing the path to *in-vivo* imaging in freely moving mice via multimode-multicore fiber based holographic endoscopy

**DOI:** 10.1117/1.NPh.11.S1.S11506

**Published:** 2024-02-13

**Authors:** Yang Du, Evelyn Dylda, Miroslav Stibůrek, André D Gomes, Sergey Turtaev, Janelle M. P. Pakan, Tomáš Čižmár

**Affiliations:** a University of Chinese Academy of Sciences, Hangzhou Institute for Advanced Study, Hangzhou, China; b Leibniz Institute of Photonic Technology, Jena, Germany; c Otto-von-Guericke-University Magdeburg, Institute of Cognitive Neurology and Dementia Research, Magdeburg, Germany; d Center for Behavioral Brain Sciences, Magdeburg, Germany; e Institute of Scientific Instruments of CAS, Brno, Czechia; f German Centre for Neurodegenerative Diseases, Magdeburg, Germany; g Leibniz Institute for Neurobiology, Magdeburg, Germany; h Friedrich Schiller University Jena, Institute of Applied Optics, Jena, Germany

**Keywords:** multimode fiber, multicore fiber, *in-vivo* imaging, freely moving, fiber connection, holographic endoscopy

## Abstract

**Significance:**

Hair-thin multimode optical fiber-based holographic endoscopes have gained considerable interest in modern neuroscience for their ability to achieve cellular and even subcellular resolution during *in-vivo* deep brain imaging. However, the application of multimode fibers in freely moving animals presents a persistent challenge as it is difficult to maintain optimal imaging performance while the fiber undergoes deformations.

**Aim:**

We propose a fiber solution for challenging *in-vivo* applications with the capability of deep brain high spatial resolution imaging and neuronal activity monitoring in anesthetized as well as awake behaving mice.

**Approach:**

We used our previously developed $M^{3}\mathrm{CF}$ multimode-multicore fiber to record fluorescently labeled neurons in anesthetized mice. Our $M^{3}\mathrm{CF}$ exhibits a cascaded refractive index structure, enabling two distinct regimes of light transport that imitate either a multimode or a multicore fiber. The $M^{3}\mathrm{CF}$ has been specifically designed for use in the initial phase of an *in-vivo* experiment, allowing for the navigation of the endoscope’s distal end toward the targeted brain structure. The multicore regime enables the transfer of light to and from each individual neuron within the field of view. For chronic experiments in awake behaving mice, it is crucial to allow for disconnecting the fiber and the animal between experiments. Therefore, we provide here an effective solution and establish a protocol for reconnection of two segments of $M^{3}\mathrm{CF}$ with hexagonally arranged corelets.

**Results:**

We successfully utilized the $M^{3}\mathrm{CF}$ to image neurons in anaesthetized transgenic mice expressing enhanced green fluorescent protein. Additionally, we compared imaging results obtained with the $M^{3}\mathrm{CF}$ with larger numerical aperture (NA) fibers in fixed whole-brain tissue.

**Conclusions:**

This study focuses on addressing challenges and providing insights into the use of multimode-multicore fibers as imaging solutions for *in-vivo* applications. We suggest that the upcoming version of the $M^{3}\mathrm{CF}$ increases the overall NA between the two cladding layers to allow for access to high resolution spatial imaging. As the NA increases in the multimode regime, the fiber diameter and ring structure must be reduced to minimize the computational burden and invasiveness.

## 1 Introduction

Neuroscience has witnessed a pivotal development with the utilization of optical toolsets to elucidate the relationship between brain function and behavior.^1^^–^^5^ Multiphoton microscopy, a widely used imaging technique,^6^^–^^10^ has facilitated this goal by enabling *in-vivo* monitoring of neuronal activity with high spatial and temporal resolution, even down to the single-cell level, in awake head-fixed mice. However, head-fixed animals are restricted in their natural behaviors. Thus, alternative imaging approaches, such as flexible two-photon fiberscopes,^11^^–^^14^ have been developed to allow for activity monitoring in freely moving mice. Nevertheless, light delivery to deep brain regions still poses a significant challenge due to tissue scattering, which impairs optical power focus beyond ∼1  mm. Deep brain imaging solutions such as the use of gradient-index (GRIN) lenses overcome this problem as the lens enables the light to travel without scattering. However, the implantation of a GRIN lens (often up to 1 mm in diameter) into brain tissue causes severe brain damage and alters neuronal connections and, thus, patterns of neuronal activity. Optical fibers, such as multi-channel probes,^15^ fiber bundles,^16^ and tapered fibers,^17^^,^^18^ have emerged as an effective solution for light delivery and neural activity recording in deep brain regions due to their small size and flexibility.

Modern hair-thin multimode optical fiber-based holographic endoscopes have gained significant attention due to their ability to successfully achieve *in-vivo* deep brain imaging^19^^–^^21^ with cellular and even subcellular resolution. A recent study conducted by Stibůrek et al.^22^ demonstrated a groundbreaking achievement of whole-depth brain imaging, with depths of up to 4 mm, using a side-polished multimode fiber probe. However, a challenge that still persists is the application of multimode fibers in freely moving animals as fibers do not maintain the highest level of imaging performance while undergoing deformations.^23^^–^^27^ To address this issue, various strategies have been proposed; these include the application of complex numerical methods and machine learning-assisted algorithms,^28^^,^^29^ the utilization of guide stars,^30^^,^^31^ and the bending of resilient fibers.^32^^,^^33^ It is important to note that the ultimate goal of achieving high-resolution separate stimulation within a field of view of the fiber necessitates the use of multicore fibers. This is because multicore fibers facilitate light transport to and from each individual core, i.e., at the spatial resolution of a single neuron, despite the motion of a behaving animal. Therefore, making this technology applicable in freely moving mice is a crucial step toward realizing this goal.

In our previous work,^34^ we presented a novel hybrid multimode-multicore fiber ($M^{3}\mathrm{CF}$) with 61 corelets. This fiber features a cascaded refractive index structure and allows for two distinct regimes of light transport that mimic either a multimode or a multicore fiber. The intended application of the $M^{3}\mathrm{CF}$ is for use in the initial phase of an *in-vivo* experiment to navigate the distal end of the endoscope toward a targeted brain structure and map the layout and connectivity of neurons within the circuit being investigated in anaesthetized mice using the multimode setting of the fiber. After implantation and anchoring of the fiber, the multicore regime facilitates light transport to and from each individual core (i.e., at the spatial resolution of a single neuron) within the field of view, despite the motion of a behaving animal. Therefore, our proposed application scenario comprises two parts: an acute high-resolution imaging session (multimode regime) for guided implantation in sedated animals and chronic studies in the awake behaving animal utilizing low-resolution (multicore regime) stimulation and readout of pre-selected areas of interest.

In this study, we focus on *in-vivo* experiments utilizing the hybrid $M^{3}\mathrm{CF}$ fiber and examine the optical properties, with a particular focus on its performance under deformation. Specifically, we achieved successful imaging of the cellular structures of neurons in anaesthetized transgenic mice expressing enhanced green fluorescent protein (eGFP) throughout the depths of the cerebellum, a traditionally difficult brain structure to access with optical methods. We provided an effective solution for connecting two hexagonally arranged corelets in the $M^{3}\mathrm{CF}$ and established a protocol for reconnecting the corelets to enable chronic studies in the future. Furthermore, we compared imaging results obtained in fixed brain tissue with other relatively large numerical aperture (NA) fibers in comparison with the $M^{3}\mathrm{CF}$. Additionally, the use of the $M^{3}\mathrm{CF}$ enables imaging of larger cell populations (maximum field of view Ø230  μm). We anticipate that an NA-enlarged $M^{3}\mathrm{CF}$ could provide the neuroscience community a novel solution for studying large neuronal populations in the deepest brain regions.

## 2 Methods

### 2.1 Multimode-Multicore Fiber Imaging Setup

**Fig. 1:**
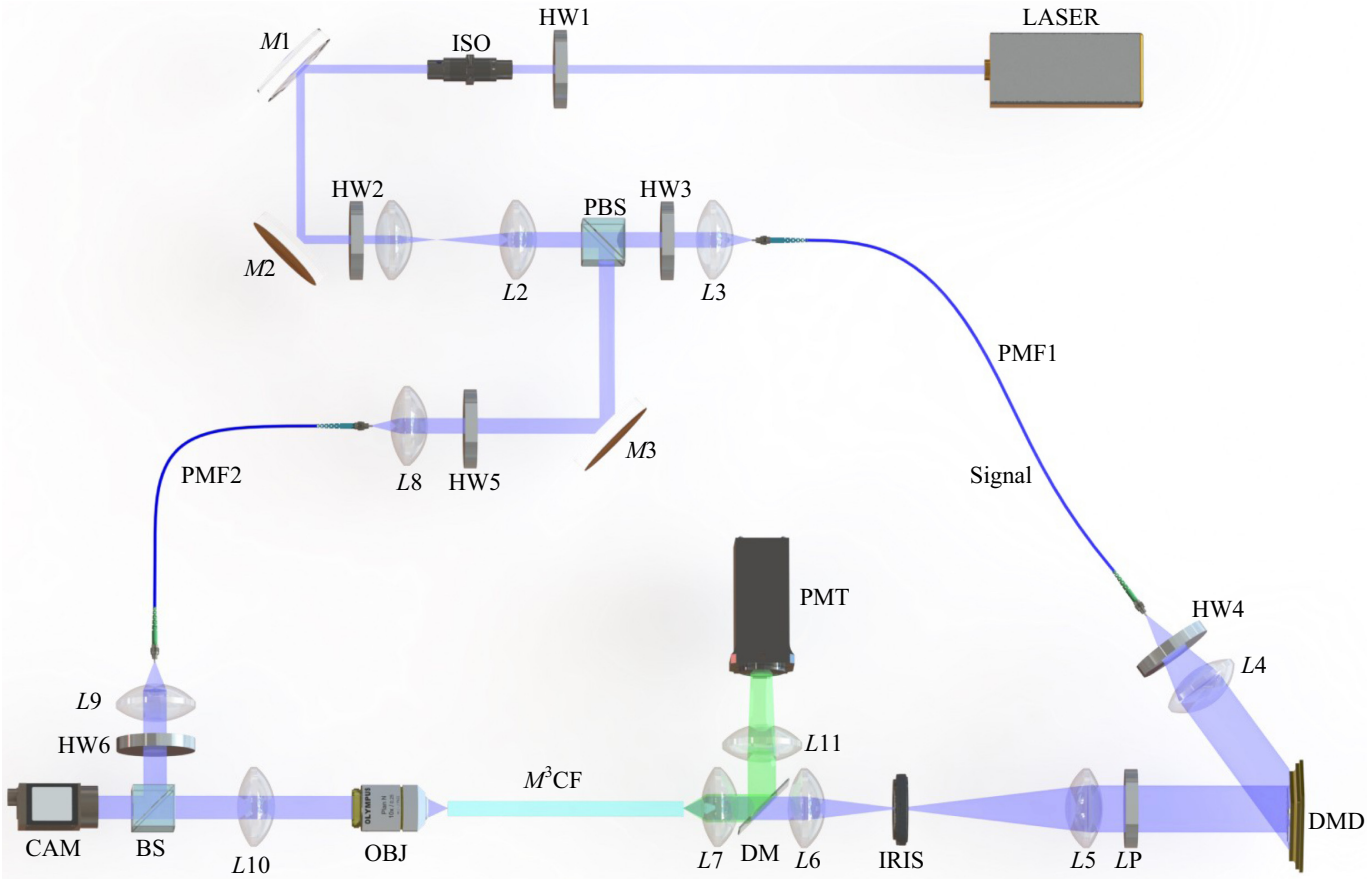
Scheme of multicore fiber-based imaging setup. LASER: Coherent Sapphire SF 488; HW1-HW6: Thorlabs WPH05M-488; ISO: Thorlabs IO-3-488-HP; M1-M3: Thorlabs BB1-E02; L1: Thorlabs AC254-050-A-ML; L2, L4: Thorlabs AC254-100-A-ML; L3, L7, L8, L9: Thorlabs A240TM-A; L5: Thorlabs AC254-200-A-ML; L6: Thorlabs AC254-200-A-ML; L10: Thorlabs AC254-125-A-ML;L11: Thorlabs AC254-60-A-ML; DM: Thorlabs MD498; PMT: Thorlabs PMT2101; PMF1: Thorlabs P5-488PM-FC-1; PMF2: Thorlabs P5-488PM-FC-2; DMD: ViALUX V7001; LP: Thorlabs LPVISE100-A; IRIS: Thorlabs SM2D25D; OBJ: Olympus Plan Achromat 10X NA 0.25; PBS: Thorlabs PBS101; BS: Thorlabs BS010; and CAM: Basler acA640-750  μm. Beam-blocks and neutral-density filters are not shown in the scheme.

The experimental setup is illustrated in Fig. 1. A single frequency continuous wave laser at a wavelength of 488 nm was used in all experiments. Linearly polarized light beam from the laser is expanded by a telescope formed from two achromatic lenses L1 and L2. The light beam is separated by a polarizing beam splitter (PBS) to a signal and a reference beam. A half wave plate HW1 and an optical isolator (ISO) are added to control the overall power of the two beams. A half wave plate HW2 is inserted to alter the power ratio between the two beams. These two beams are further coupled into single mode polarization maintaining fibers PMF1 and PMF2 via achromatic lenses L3 and L8, respectively.

Half wave plates HW3 and HW5 align the two beams with either a fast or slow axis of the polarization-maintaining fibers. A half wave plate HW4, together with a linear polarizer LP are applied to ensure a linearly polarized light to be coupled into the $M^{3}\mathrm{CF}$ fiber. In the signal path, the beam is collimated by an achromatic lens L4 to overfill the active area of a digital micromirror device (DMD). The DMD is installed in an off-axis regime at an angle of 24 deg with respect to the incident light beam. The phase of incident light on the DMD is controlled using binary-amplitude gratings. At the Fourier plane of the DMD, after lens L5, the first-order diffracted signal is isolated using an iris and is relayed by lenses L6 and L7 to the proximal end of $M^{3}\mathrm{CF}$. The focal lengths of L5, L6, and L7 are carefully selected to adapt to the NA of $M^{3}\mathrm{CF}$. Microscope objective lens OBJ and tube lens L10 image the distal end of fiber facet onto a complementary metal-oxide-semiconductor (CMOS) camera. In the reference path, a lens L9 collimates the beam exiting from PMF2. A half wave plate HW6 is chosen to adjust the linear polarization angle of the reference beam for maximizing the contrast of the interference signal. A non-polarizing beam splitter BS is placed to combine the signal and the reference beams.

We adopt the transmission matrix (TM) algorithm for controlling the light transport through the $M^{3}\mathrm{CF}$ and image acquisition.^35^^,^^36^ The TM represents a linear relationship between input and output optical modes of a given medium, and with its availability, any desired output field can be synthesized despite the medium’s complexity. The DMD (chip size: 1024×768  pixels) is a programmable component employed in the system for light beam shaping through the fiber. Although DMD is a binary amplitude light modulator, it enables providing phase modulation when it is applied in the off-axis regime. The $M^{3}\mathrm{CF}$ supports approximately 12500 modes at the wavelength of 488 nm. The input modes are chosen as a square grid of 201×201 diffraction-limited foci. Prior to the TM acquisition, a primary alignment is performed by integrating the output field received from the camera for each individual input mode. This step ensures that the fiber is correctly positioned and oriented with respect to the imaging system, and it eliminates any input modes falling outside the spatial constraints of the fiber, reducing their number to ∼26000. The DMD utilized in our experiment has an onboard RAM of 16 GB and supports a maximum of 174,762 patterns for scanning. The extensive use of input modes, nearing the hardware’s maximum capacity, was necessitated by the substantial core dimension of the fiber. The entire calibration process was completed in about 3 min using a standard laboratory desktop computer equipped with an Intel i7-6700 3.4 GHz processor and 64 GB RAM. Both the calibration and pattern generation are performed before each imaging session. Once the sequence of holograms is calculated, the imaging session starts. At the distal end of the fiber, the output modes are analogously occupying a square grid of 352×352 focal points with an interval of ∼0.7  μm to fully cover and significantly oversample the entire $M^{3}\mathrm{CF}$ core during acquisition. Further, during image acquisition, the number of output modes is analogously reduced to ∼87000 because only these fall into the circular area of the core. The TM is acquired using phase-shift interferometry and is used to generate diffraction-limited foci at the $M^{3}\mathrm{CF}$ output, as described in detail in our previous work.^34^ After the TM acquisition, the calibration module at the fiber distal end is removed and replaced by the mouse.

### 2.2 Animals and Experimental Preparation

All animal experiments were approved by the animal care committee of Sachsen-Anhalt, Germany, and conform with the European Directive 86/609/EEC and 2010/63/EU on the protection of animals used for experimental purposes. Mice were group housed (typically 2 to 4 mice), and both male and female adult mice aged 24 to 57 weeks were used. Mice were housed in standard cages at a 12h/12h light dark cycle, and food and water were provided *ad libitum*. For *in vivo* imaging experiments in anaesthetized mice, three female mice expressing eGFP in a subset of cerebellar Purkinje cells driven by an EAAT4 promoter (EAAT4 mice^37^) were used.

Mice were anaesthetized with isoflurane (4% for induction and 1% to 2% maintenance during surgery) and mounted in a stereotaxic frame (Stoelting). Eye cream was applied to protect the eyes from dehydration and light (Bepanthen, Bayer), and analgesics and anti-inflammatory drugs were injected subcutaneously (buprenorphine, 0.1  mg/kg of body weight, carprofen, 0.15 mg, and dexamethasone, 2  μg). A section of the scalp was removed, and a craniotomy was made exposing the cerebellum (posterior to lambda). The cavity was filled with phosphate-buffered saline (PBS), and the mouse was moved to a small stereotaxic frame below the imaging fiber for the duration of imaging before being transcardially purfused. Additional EAAT4 mice were used for fixed whole-brain imaging following transcardial perfusion. For perfusions, animals were injected with a lethal overdose of sodium pentobarbitol (400  mg/kg) and transcardially perfused with 0.9% saline followed by 4% PFA in phosphate buffer saline (0.1 M PBS).

## 3 Results

### 3.1 Multimode-Multicore Fibers

**Fig. 2:**
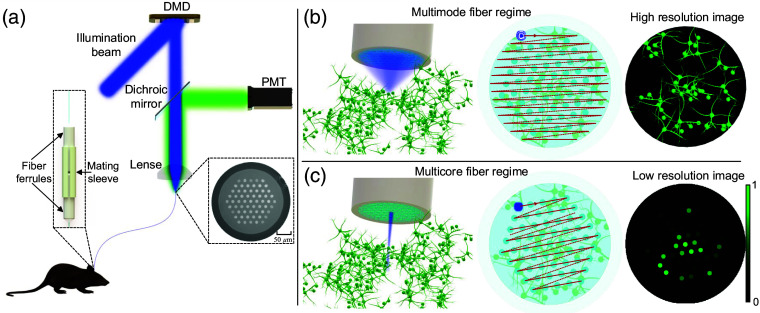
Imaging mechanism of multimode-multicore fiber. (a) Schematics of the imaging setup. Inset left side: connection method for two pieces of fiber. Inset right side: microscope image of the cross-section of the $M^{3}\mathrm{CF}$. (b) Illustration of $M^{3}\mathrm{CF}$ working under the multimode regime. (c) Illustration of $M^{3}\mathrm{CF}$ working under the multicore regime (Video 1, MPEG, 5.54 MB [URL: https://doi.org/10.1117/1.NPh.11.S1.S11506.s1]).

In our work, we integrated an optical fiber in a holographic endoscopy imaging system to generate diffraction-limited focal spots at any desired position across the distal fiber facet. A detailed implementation of this setup can be found in the Methods section, and a schematic imaging setup is presented in Fig. 2(a). The use of a DMD placed in a far-field plane to the input fiber facet allows for the projection of various holograms on the DMD to achieve different output fields. The fiber consists of two pieces: one short piece that is implanted chronically into the brain of an animal and a long fiber that is connected to the imaging setup. Both fibers are connected at a mating point [Fig. 2(a)].

The cross-section of the multimode-multicore fiber [$M^{3}\mathrm{CF}$, Fig. 2(a)] consists of 61 corelets, as reported previously.^34^ The overall cladding and pulp, with diameters of 278  μm and 232  μm, respectively, form a step-index multimode waveguide with a refractive index difference corresponding to an NA of 0.1. In the multimode regime, as shown in Fig. 2(b), we utilize the fiber TM method to generate diffraction-limited focal points. After characterizing the fiber, we insert the fiber probe into the brain of a transgenic mouse for *in-vivo* imaging. During imaging, we raster scan the focal points across the entire fiber facet to obtain a high-resolution image of neuronal populations. For the potential option of chronic *in-vivo* imaging, it could be possible that, once the high-resolution image is achieved, the inserted fiber probe might be fixed to the skull of the animal. This would potentially allow the mouse to move and behave freely while the fiber operates under the multicore regime, as illustrated in Fig. 2(c). These corelets are deformation-resilient and can be used to either form a low-resolution image or monitor activity for any selected location within the field-of-view.

### 3.2 Fibers Connection

A critical aspect of chronically recording optical signals is the availability of a reconnectable optical fiber probe. To achieve this objective, we propose a simplified connection approach between two $M^{3}\mathrm{CF}s$. Unlike conventional optical fibers, these fibers typically feature a solitary core, and rotational considerations are not an issue when joining two segments. As depicted in Fig. 2(a), our $M^{3}\mathrm{CF}$ features 61 corelets are arranged in a hexagonal pattern. Consequently, a high-precision protocol must be employed to achieve a seamless connection between all 61 corelets.

In Fig. 2(a), as depicted in the left inset picture, two endface-cleaved fibers are bonded using UV curing adhesive (NOA68, Norland Products Inc.) within fiber ferrules. Prior to curing, both endfaces are aligned with their respective ferrule facets. The shorter fiber piece, with a tip length of approximately ∼4  mm, protrudes from the ferrule and is intended for implantation in the mouse brain after the initial imaging phase. To minimize lateral misalignments during reconnection, customized fiber ferrules (Femotech GmbH) with an inner bore size of 280±3  μm and a standard outer diameter of 2.5 mm were selected; this accommodates our $M^{3}\mathrm{CF}$ outer diameter of 278  μm. To ensure a secure connection, two fiber ferrules are tightly mated using a ferrule mating sleeve (ADAF1, Thorlabs) with a ferrule adapting diameter of 2.5 mm. This mating sleeve enables the quick release of the longer fiber piece while maintaining an adequate mating tightness between ferrules. To further elucidate the degree of connection precision, a sequence of focal points was generated at the far field of the fiber. Following the calibration of two connected fibers, the scanning points were projected onto a white surface to assess the alignment. As a result of the good alignment precision of the fiber mating, the observation of the same scanning points was achievable subsequent to re-connection (Video 1).

**Fig. 3:**
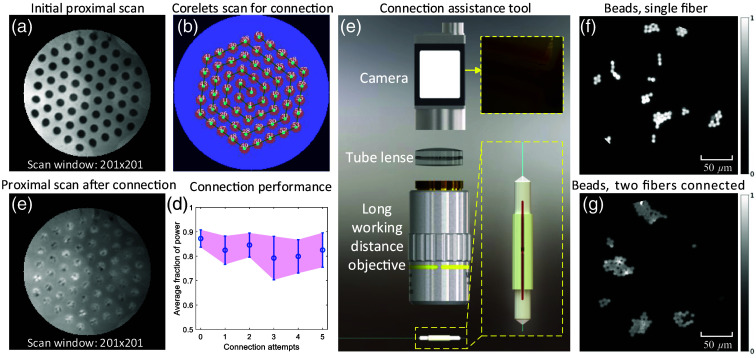
Precise connection of two $M^{3}\mathrm{CF}s$. (a) Integrated image via scan across a single fiber facet at its proximal end. (b) Scan sequence across corelets arranged in a spiral manner. (c) Integrated image via scan across two connected fiber facets at its proximal end. (d) Averaged fraction of power preserved in the addressed corelets at various re-connection attempts. (e) A compact assistance tool for re-connection by comparing image similarities of the marker region between the initial and re-connected conditions. (f), (g) Images of fluorescent beads measured with a single fiber and two connected fibers, respectively.

Prior to connecting two fibers, light was coupled at the proximal end of the first fiber, and a camera was placed at the fiber distal end to align the fibers. To ensure sufficient scanning of the input modes and resemble an accurate proximal fiber map, holograms with a scan window of 201×201 were applied, taking into account the number of modes that the fiber supported. The integration of all scanned images produced an integrated proximal end image, as depicted in Fig. 3(a), from which holograms targeting any desired proximal positions could be selected based on their 2D coordinates. These selected holograms were sorted in a spiral scan sequence for light coupling only into the corelets [Fig. 3(b)]. It should be noted that these holograms could also be used for low-resolution imaging or activity monitoring at the desired corelet positions. Once the two fibers were mated using a mating sleeve [as shown in Fig. 3(e)], the selected holograms served as a light guide for the fiber connection, and a camera was placed at the distal end of the second fiber. By rotating the first fiber ferrule until sufficient light coupling to all corelets of the second fiber was observed, a typical proximal scan integration was obtained [Fig. 3(c)]. However, the light distribution within the corelets was non-uniform due to fiber-to-fiber coupling mismatch. Nevertheless, all corelets were observable and could be addressed individually during testing. To assess the quality of the fiber connection, we measured the light power for each corelet over the entire fiber facet by recording high dynamic range images of the output fiber facet while coupling light to individual corelets. The average fraction power of all corelets was then plotted [Fig. 3(d)], with connection attempt “0” representing the initial single-fiber case. The results of five reconnection attempts are shown in Fig. 3(d). After reconnection, the average power fraction only drops, on average, by 5%.

Re-establishing connections between fibers is facilitated by employing an external imaging tool [Fig. 3(e)]. One important consideration in this process is the loss of access to the distal end of the fiber facet once the fiber distal end is implanted into the mouse brain. To overcome this challenge, we affixed a marker to both ferrules, as shown in Fig. 3(e), for future reference after successfully connecting the corelets of the fibers. Subsequently, an external compact microscope, consisting of a long working distance objective (10×, Mitutoyo), a tube lens (AC254-060-A-ML, Thorlabs), and a color camera (acA720-520uc, Basler), was utilized to observe the marker region. An image was taken after the initial connection, and subsequent images were captured during reconnection to ensure optimal connection performance.

A comparison of imaging performance for fiber probes is presented in Figs. 3(f) and 3(g), which showcase high-resolution images of fluorescent beads with a diameter of 6  μm (Fluoresbrite YG Microspheres, Polysciences Europe GmbH) between a single fiber and two connected fibers. These images have been normalized to the same scale. The contrast in the image acquired with a connected fiber is slightly lower than the contrast obtained with a single fiber. This can be attributed to the degradation of the power ratio in a complex waveguide structure (fiber-air gap-fiber) compared with a single fiber. Notably, all subsequent measurements presented are obtained using a connected and calibrated fiber. Connection and calibration of the fiber was performed before the fiber was lowered into the brain of an anesthetized mouse.

### 3.3 *In-Vivo* Imaging with $M^{3}\mathrm{CF}$

**Fig. 4:**
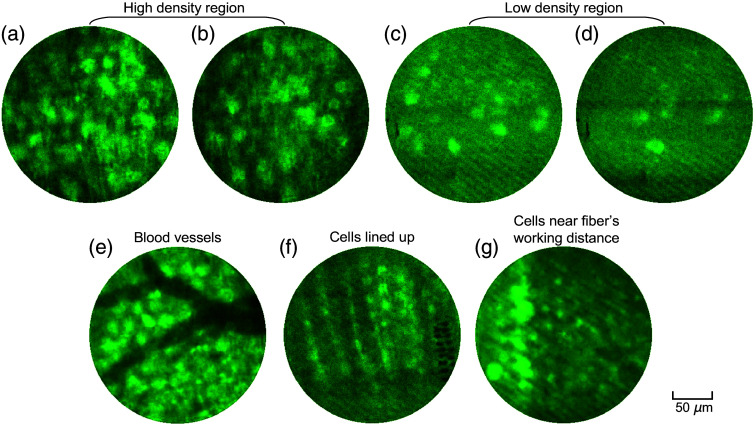
*In-vivo* imaging with $M^{3}\mathrm{CF}s$. Images of Purkinje cells in the cerebellum of transgenic EAAT4 mice observed during multiple insertions of the $M^{3}\mathrm{CF}$. (a), (b) Labeled Purkinje cell soma imaged using the $M^{3}\mathrm{CF}$ fiber within cells high density regions of the cerebellar cortex. (c), (d) Labeled Purkinje cell somas imaged using the $M^{3}\mathrm{CF}$ fiber within cells’ low-density regions of the cerebellar cortex. (e) Image of identified blood vessels within the tissue during the fiber insertion. (f) Long thin processes image from single layer within the cerebellar cortex. (g) Purkinje cell soma imaged at near the fiber’s effective working distance (Video 2, MPEG, 5.38 MB [URL: https://doi.org/10.1117/1.NPh.11.S1.S11506.s2]) (Video 3, MPEG, 4.18 MB [URL: https://doi.org/10.1117/1.NPh.11.S1.S11506.s3]).

To demonstrate the imaging capabilities of the $M^{3}\mathrm{CF}$ in animal models, we conducted *in-vivo* experiments on transgenic EAAT4 mice in which a subset of Purkinje cells throughout the depths of the cerebellar cortex are labeled with eGFP.^37^ The fiber probe was inserted into the cerebellum at depths of up to ∼3  mm from the brain surface, and we captured structural images during various insertions of the fiber in different regions of the tissue (Fig. 4). Labeled Purkinje cell somas imaged using the $M^{3}\mathrm{CF}$ fiber within various regions of the cerebellar cortex in the multimode regime are shown in Figs. 4(a)–4(g). Figure 4(e) additionally shows imaging of identified blood vessels within the tissue (dark structures). The dynamic insertion process is clearly demonstrated in Video 2, and imaging of eGFP labeled cell bodies is shown in Video 3. Figure 4(f) presents an image of Purkinje cells distinguishable in a single layer formation within the cerebellar cortex. Additionally, longer thin processes can be seen in some images that have the stereotypical structure of Purkinje cell dendrites that also extend from this single layer within the cerebellar cortex [Fig. 4(f)]. For our *in-vivo* imaging experiments, we set the fiber’s effective working distance at 15  μm, i.e., the distance between the imaging plane of the calibration camera and the fiber facet. It was noted that motion artifacts could sometimes be seen in the acquired images, which can be attributed to movement artefacts originating from the heart beating and breathing. Therefore, our successful demonstration of *in-vivo* structural imaging utilizing static eGFP labeling in anesthetized mice provided valuable insight into the physical properties of our $M^{3}\mathrm{CF}$ fiber for chronic *in-vivo* applications in living animals.

### 3.4 Imaging Comparison with Larger NA Fibers

**Fig. 5:**
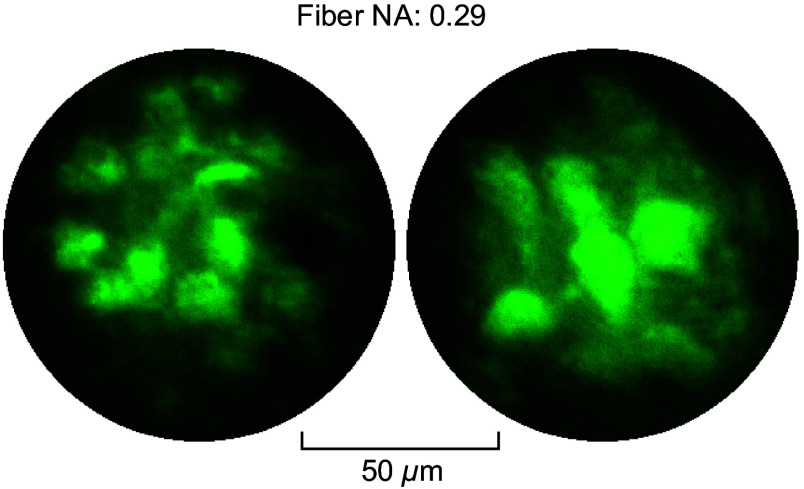
*In-vivo* imaging with a larger NA fiber. Images of Purkinje cells in the cerebellum of transgenic EAAT4 mice observed during multiple insertions of the fiber probe.

We also conducted an examination using a higher NA fiber (GI2016-F, Yangtze Optical FC, China), characterized by a core diameter of 100  μm, a cladding diameter of 125  μm, and an NA of 0.29, which was inserted into the cerebellum of a transgenic EAAT4 mouse. The utilization of a fiber with a higher NA facilitated the acquisition of images of densely packed Purkinje cells with an improved contrast, as demonstrated in Fig. 5.

**Fig. 6:**
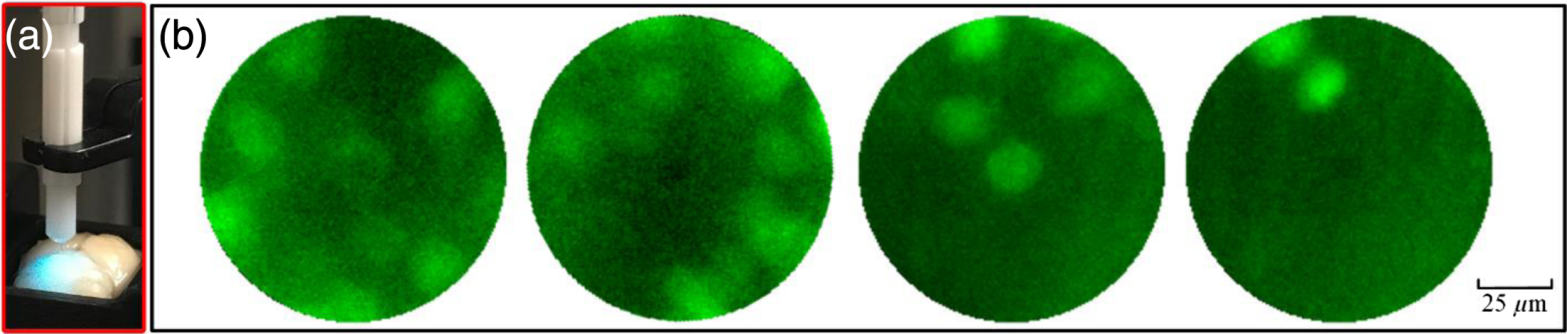
Imaging with a double-clad fiber (DCF, NA: 0.20) in fixed whole-brain tissue. (a) Brain tissue was submerged within a phosphate buffered saline (0.1 M PBS) solution in a container, and the fiber probe was constructed using two pieces of fiber that could be disconnected and reconnected. (b) Images of Purkinje cells in the cerebellum of transgenic EAAT4 mice were observed during multiple insertions of the DCF probe.

We further performed a test of the imaging performance of a double-clad fiber (DCF13, Thorlabs) in fixed whole-brain tissue. The DCF has a larger NA (0.20) of the multimode structure than the $M^{3}\mathrm{CF}$ (0.10) used in the previous experiment. The double-clad fiber shares similarities with our $M^{3}\mathrm{CF}$, featuring a single mode core and dual cladding structure that permits both single mode and multimode light to propagate through the fiber. Specifically, single mode light travels through the 9  μm core, whereas multimode light propagates through a 105  μm inner “1st cladding.” The fiber’s outer diameter is 125  μm. The DCF was used to image deep within the fixed transgenic EAAT4 mouse whole-brain tissue. The brain was placed in a container and covered with PBS solution. The fiber probe consists of two pieces of fibers constructed in the same manner as the $M^{3}\mathrm{CF}$ probe [Fig. 6(a)]. The imaging results with the DCF, demonstrated in Fig. 6(b), show spatially separated Purkinje cell somas in densely labeled brain tissue. The achieved imaging results show better contrast than using the $M^{3}\mathrm{CF}$.

## 4 Discussion

In this study, we delve deeper into our exploration of our previously introduced hybrid multimode-multicore fiber ($M^{3}\mathrm{CF}$) by investigating the possibilities and challenges *in vivo* and according to various fiber parameters. The $M^{3}\mathrm{CF}$ architecture is designed to cater for both multimode and multicore imaging regimes. In the multimode regime, the fiber remains stationary post-calibration and was used for *in-vivo* imaging in anesthetized mice, enabling visualization of neurons within a field of view of Ø230  μm. Subsequently, the $M^{3}\mathrm{CF}$ can be utilized in the multicore regime for low-resolution imaging. The switch to the low-resolution multicore regime is necessary to address individual corelets’ overlapped neuron cells for imaging/monitoring while the fiber is under deformation.

We successfully implemented the $M^{3}\mathrm{CF}$ for *in-vivo* imaging in anesthetized transgenic eGFP expressing mice, demonstrating its structural imaging capabilities. However, the images produced by the $M^{3}\mathrm{CF}$ displayed low contrast, particularly in densely labeled brain tissue. The achieved spatial resolution of 2  μm was constrained by the NA of the fiber (0.10). During the insertion process of the fiber into the mouse brain, we observed tissue displacements due to the relatively large diameter (280  μm) of the $M^{3}\mathrm{CF}$ when compared with most commonly used single-core multimodal fibers.^21^ This issue could be mitigated by producing a fiber with a smaller diameter and an angled fiber tip. Note that imaging artefacts can be observed due to the presence of 61 corelets. This is caused by the cascaded refractive index fiber profile, as we explained in our previous work.^34^ Consequently, light appears brighter in the corelets than in the pulp region. To achieve light uniformity in these regions, we suggest employing methods such as measuring the fiber’s TM to normalize the foci in the corelets region to those in the pulp or using different interference signals to measure separate transmission matrices for the corelets and pulp areas.

In this study, we provided insights and identified challenges in utilizing multimode-multicore fibers as imaging-assisted spatial monitoring solutions for *in-vivo* applications. We compared the imaging performance of the $M^{3}\mathrm{CF}$ with a standard graded index fiber and single corelet double clad fiber but with a larger NA. We suggest that the next iteration of $M^{3}\mathrm{CF}$ increases the overall NA between the two cladding layers to achieve better imaging spatial resolution and fluorescent collection efficiency. This increase in the NA is intended solely for the multimode regime, and we plan to maintain the NA of the corelets unchanged to ensure minimal crosstalk. In practical optical fiber fabrication, the availability of suitable materials for achieving the desired NA is also a crucial consideration. However, as the NA increases in the multimode regime, it is necessary to reduce the fiber diameter and ring structure. This reduction is important for minimizing the computational burden and invasiveness as the overall mode number supported in the fiber increases significantly. We plan to design the fiber with specific applications in mind, tailoring it to the desired field of view and the number of neurons required to be imaged. Our vision for future optimized $M^{3}\mathrm{CF}$ probes is to enable researchers in the neuroscience community to access the deepest brain regions while using the most advanced neurophotonics tools to study neuronal activity.

## Supplementary Material







## Data Availability

All relevant raw datasets generated and analyzed during the studies presented in this article can be found in the Zenodo repository, https://zenodo.org/records/10521980.
